# Blockchain localization spoofing detection based on fuzzy AHP in IoT systems

**DOI:** 10.1186/s13638-022-02094-7

**Published:** 2022-03-02

**Authors:** Wenzhe Lv, Xuesong Qiu, Luoming Meng

**Affiliations:** State Key Laboratory of Networking and Switching Technology, Beijing University of Posts and Telecommunications, Beijing, China

**Keywords:** Location spoofing detection, Fuzzy analytic hierarchy process, Blockchain

## Abstract

Location spoof detection is a major component of location proofing mechanisms in internet of things (IoT), and it is significant for the system to assess the trustworthiness of the location data associated with the user. Unlike the work that employs physical layer features, we interest in building the infrastructure for a solution to establish location spoofing detection capabilities in blockchain-based IoT systems. In detail, at the node and the mobile trajectory level, we create an IoT system for evaluating the trustworthiness of location proofs with blockchain location system features. A blockchain-based multilayer fuzzy hierarchical analysis process (AHP) evaluation method is contemplated to detect location spoofing in the IoT system. Simulation results indicate the proposed method has a superior performance and provides a basis for the trustworthiness assessment of location proofs.

## Introduction

With the rapid development of artificial intelligence (AI) and industrial technology, internet of things (IoT) has become an emerging research field. IoT is an important cornerstone of the fourth industrial revolution and a key measure to transform old kinetic energy into a new one.

Meanwhile, contact tracing has been a hot issue with the rapid spread of COVID-19 all around the world. Many location-based services (LBS) and activity-tracking applications (ATA) with short-range communication (SRC) techniques are studied in depth for application, especially for automated contact tracing [1]. However, many individuals are wary of sharing location or contact data, as well as private data, with technology companies or governments, how to protect the privacy of data is an essential problem to be solved in the IoT systems.

As a solution to solve the issues of privacy, security, and deployment efficiency in automated contact tracing and proof of location (PoL), the Blockchain technology has attracted more attention in IoT [2]. The blockchain system assumes that the location information submitted by the prover to the blockchain is reliable when it is used for contact tracking of COVID-19 or other task scenarios that require accurate location positioning.

Unfortunately, if location-based activities provide an incentive mechanism to encourage users to participate, malicious users may change their location programmatically. They thus obtain unfair advantages and rewards by providing a fake location to the system. This behavior of users providing a wrong location is known as location spoofing attack (LSA) [3]. Without totally authentic and trustworthy information, location-based services in IoT can cause inconvenience to users, and even serious harm in specific scenarios, such as driving. Due to the interests that will aggravate the emergence of this behavior, the detection of location spoofing in IoT attempts has become increasingly important. Although using LSA can bring unfair benefits to attackers, it is at the cost of interfering with the common operation of system and the quality of service. Therefore, it is necessary to make reliable and trustworthy estimates of user locations in the blockchain systems.

To address this issue, different distributed PoL schemes have been proposed to detect location spoofing attacks from dishonest users. Wang et al. [4] proposed a secure and privacy-aware distributed proof-of-location scheme for mobile users, named SPARSE, which provides secure and private LP generation and verification for mobile users. By limiting the interaction time between the verifier and the witness, and using a random witness selection mechanism, SPARSE can resist malicious users colluding to forge location information. The simulation results demonstrated that the success rate of the scheme exceeds 98%. Using the concept of audibility, Nosouhi et al. [5] developed an enhanced location spoofing detection algorithm (ELSA) to detect location-spoofing attacks. ELSA is based on a statistical decision theory, and leverages the received TOA delay between a user’s device and the anchors to distinguish between honest target and malicious ones. Zhang et al. [6] developed the secure location of things (SLOT) framework, which redefines the location problem as a statistical nonlinear estimation problem based on audible information. The maximum likelihood estimator for the node location is obtained through a probabilistic mixture model or difference-time of-arrival model.

Although these LSA detection schemes have improved resistance to collusion attacks by Prover or Prover Witness, and shortened the proof generation time, they still have drawbacks in privacy and security, issues which most concern people. The proposed decentralized and permission less blockchain system can guarantee the security of the privacy of users, and obtain users' real-time location information through the proof of BLE witnesses [7]. This mechanism depends on cryptography to yield SRC proofs for mobile users in its vicinity, rather than using trusted third parties. The AHP algorithm can be used for LSA detection in the blockchain system. Soni [8] presented an AHP implementation to deal with the issue of trust management in the vehicular ad hoc networks (VANETs), which selects multiple indicators for trust evaluation, such as the vehicle reputation data and direct interactions. Goyal et al. [9] evaluated an AHP-based system selection policy operating under conditions in which Wi-Fi and WiMAX were operating in heterogeneous wireless networks. By analyzing the weights of the parameters, including the uplink data rate, RSSI, and power consumption, the total amount of uploaded data provided by the policy using AHP was 1.14 times higher than that of the traditional model.

In a blockchain system, the possible forms of location spoofing may include: (1) malicious users submit nodes with false locations to obtain rewards provided by the blockchain incentive mechanism; (2) to seek more benefits, dishonest users unite with a single node to cheat and upload false location information; (3) a combination of multiple nodes cheating, simulating false moving-track information by collusion. Once these behaviors are rampant in a blockchain system, the large number of false witness nodes and forged location information will affect the reliability of the system for contact tracking, and eventually, users will give up using the service because the service provided is unsatisfactory. The above-mentioned signifies that addressing LSAs in blockchain systems is paramount and inspires our work.

In this paper, we propose a scheme that efficiently and effectively tackles LSAs in the blockchain IoT system, based on the fuzzy AHP algorithm. The advantage of the scheme is gained by paying attention to the historical information of users in the blockchain system. Based on the reliability analysis of each BLE witness node in the user's action moving-track, the system judges the reliability of a verifier's action moving-track, and decides whether the current location reported by the user is credible. Simulation studies with the prototype implementation verifies the effectiveness of the scheme to identify LSAs in the blockchain system. Simulation results show that the scheme is sensitive to location spoofing, and scales well with the number of users.

The rest of the study is organized as follows. Related work part is discussed in Sect. 2. Section 3 describes the fuzzy analytical hierarchy process model and the features of the blockchain location proof. In Sect. 4, we describe the proposed scheme in depth. We present the weight calculation of the assessment index and the results of our implementations are presented in Sect. 5. Section 6 concludes the study.

## Related work

### Blockchain system

**Fig. 1:**
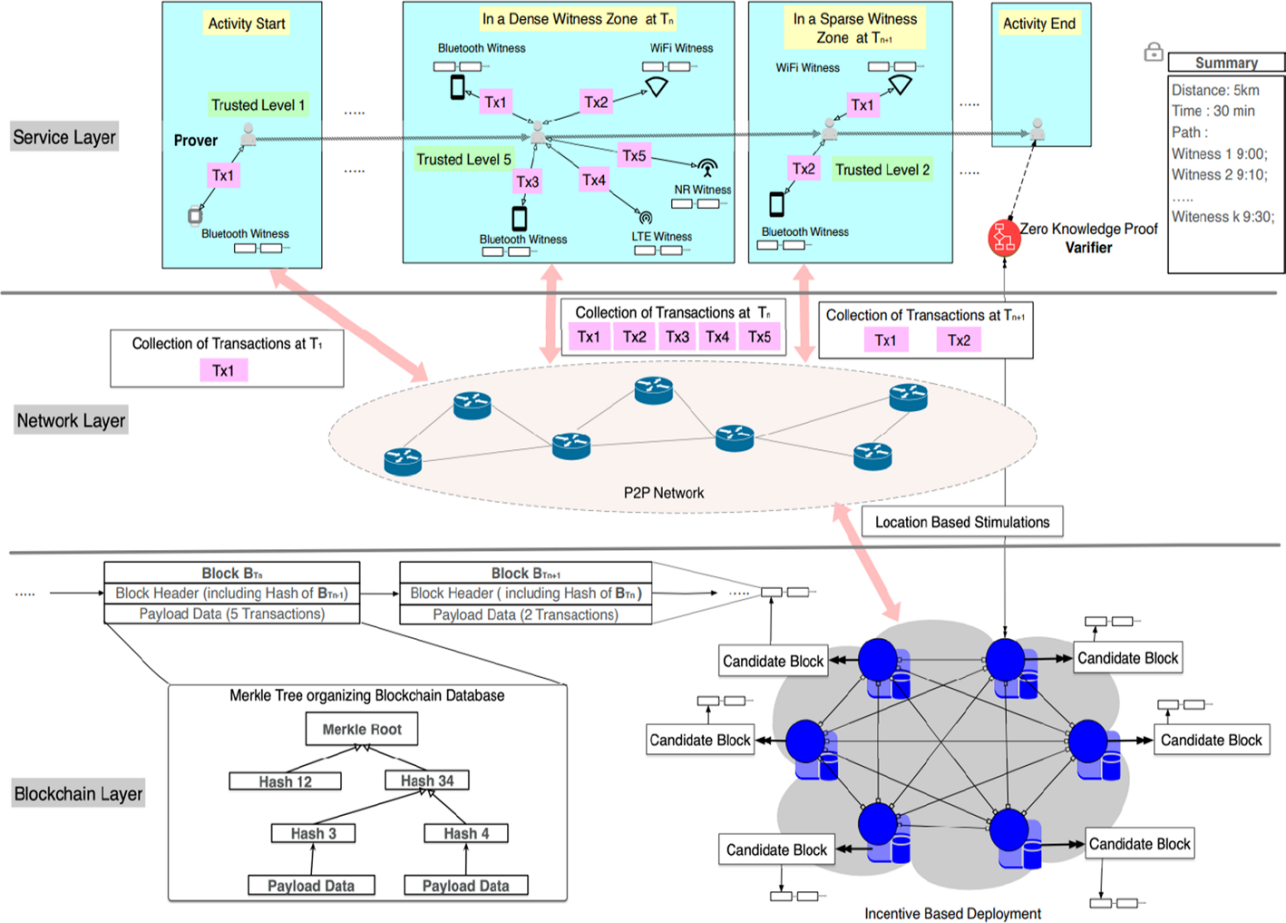
The architecture of Bychain [10]

Bychain, a blockchain system, is proposed by our research team [10], as it is shown in Fig. 1. By combining a key escrow mechanism and a zero-knowledge proof protocol, a privacy-preserving SRC protocol for activity tracking and a corresponding generalized block are generated. Firstly, wireless lightweight message exchange based on BLE technology is carried out between mobile devices and witness nodes to ensure the privacy and security of proof-of-location information. Second, proof of activity is uploaded to a decentralized ledger, which is transparent, and is distributed and saved on the server. Finally, a trustworthy summary of activity is yielded by zero computation to satisfy the COVID19 contact tracking requirement without revealing location information, which is a trustworthy summarization of the location of the authenticated user.

To realize the long-term and low consumption monitoring of high-risk hotspot areas, we choose the IoT witness based on Bluetooth low energy (BLE) technology as the nodes of the blockchain. To reduce overlap in coverage, and encourage more individuals and organizations to participate in the deployment of IoT nodes, and seek maximum monitoring coverage deployment, a virtual potential field-based incentive allocation mechanism is proposed. The incentive allocation mechanism treats each witness node is a charged particle, such that an artificial electric field is constructed in which each node is repelled by the repulsive forces of the other nodes. Thus, the witnesses in the blockchain will be dispersed throughout the environment, and will maintain force equilibrium as much as possible, to maximize rewards. In an ideal case, while witnesses are in static equilibrium and the monitoring area for blockchain network archiving is maximized.

### Location spoofing

In Bychain, there are three roles: prover, witness, and verifier. In this study, we assumed that all witnesses are low-cost, easy-to-use Bluetooth low power equipment (BLE). Each prover and witness is networked and equipped with a Global Positioning System (GPS) and Bluetooth. On the basis of smart contracts, the verifier uses interactive zero-knowledge proof, is self-running, and can protect data privacy. When the distance is low enough, witnesses can communicate with provers using SRC. The prover uploads witness-signed PoL commitments before leaving the coverage area of the witness node. Bychain releases its budget at set intervals, to incentivize IoT nodes to choose the best deployment location for maximizing the monitoring area. For the maximum reward, each node is repulsed by the repulsive forces of the other nodes. In fact, blockchain witness nodes will spread spontaneously throughout the environment to maximize the coverage of the most active areas of the city. A monitoring network based on BLE can avoid repeated coverage areas and increase the monitoring area of the blockchain to the ideal maximum.

We assume that each witness node or location service user is honest in terms of privacy in the blockchain system. The above design for privacy security ensures that the user's behavioral data cannot be leaked. However, these participants may collude with other witnesses through cheating such as LSA and collusion, to obtain improper benefits. This location spoofing mainly uses false location information to win rewards from blockchain systems or LBS event organizers. In the first case, to obtain the preset rewards provided by the blockchain system for the reasonable deployment of nodes, malicious users may claim to have deployed several BLE witness nodes required by the blockchain system. These nodes are fictitious or damaged, with non-existent node information or the wrong deployment location reported. As mentioned above, Bychain distributes incentives according to the incentive allocation scheme of the artificial virtual potential field, to motivate the nodes to coverage maximization. Only those nodes that keep an appropriate distance from the surrounding nodes in the appropriate range can get the maximum reward. However, it is difficult for users to deploy BLE nodes in any location they want in an actual environment. These limited conditions include BLE nodes that cannot be installed in private areas without permission, excessive signal interference, and other technical or subjective constraints.

Another possible situation is that the BLE witness node is arranged by trusted users in the blockchain system. However, these witness nodes may still be attacked by malicious users. Malicious users will produce false PoL messages and upload them to the chain. When there are untrusted nodes in a blockchain structure derived from the P2P mechanism, a chaotic area will inevitably appear in the working range, which will affect the normal operation of the system. Some honest users may get inaccurate or even completely wrong proof of location, while malicious users can upload the wrong POL information to the blockchain via these malicious nodes. Based on the unique challenges of the Bychain blockchain system, in this study we used a location spoofing detection system based on the fuzzy AHP algorithm to judge whether a PoL message generated by the interaction between a user and the BLE witness node is credible.

### AHP

AHP is a hierarchical weight decision analysis method proposed by American operations research expert Professor Thomas L. Saaty in the 1970s, which is used in complex multi-objective decision-making [11]. The main features of AHP are the use of a hierarchical structure, and the use of comparative judgment and synthesis of priorities for different alternatives.

AHP provides a valuable approach to the determination of the relative importance of different attributes to the objective. Establishing a hierarchical structure is the first step in implementing AHP, which makes the factors closely related to decision-making division into a target layer, criterion layer, and project level. The decision factors of the task are identified within the criterion layer. Then, a pairwise importance comparison is made between various factors in the same hierarchy, producing a comparison matrix. The relative importance can be expressed using the ranking shown in Table 1.

**Table 1:** Definition of relative importance between peer criteria

Priority	Meaning
1	The two elements are equally important
3	One element is slightly more relevant than another
5	One element is strongly more relevant than another
7	One element is very strongly more relevant than another
9	One element is extremely more relevant than another
2, 4, 6, 8	The median of 1, 3, 5, 7, 9

However, pure AHP has some shortcomings [12]. Although the original purpose of AHP was to acquire expert knowledge, the uncertainty and vagueness of subjective judgments affect the precision of the AHP ranking method. The discrete scale of 1–9 has the advantages of simplicity and ease of use, but the uncertainty associated with the mapping of decision-making judgment to priority number is not taken into account by AHP. Therefore, fuzzy set theory has been introduced into the paired comparison of AHP, to overcome these problems [13]. Fuzzy AHP methods are systematic approaches to the alternative selection and justification problem, involving integrating fuzzy set theory and hierarchical structure analysis. Fuzzy AHP can produce quantitative analysis of some problems that cannot be measured uniformly, and give a more comprehensive evaluation of the object.

The main content of this study is the use of the fuzzy AHP algorithm in the blockchain system to judge whether the location data uploaded by users is reliable, to build the blockchain location spoofing detection function.

## Methods

In this study, we used a fuzzy AHP-based approach to judge the reliability of a user’s location proof in the blockchain system.

In the blockchain system, many factors affect the credibility of the PoL message, and each factor has uncertainty and fuzziness on the effect of location spoofing detection. In this study, the main task was to quantitatively evaluate the authenticity of a user’s current location, to determine whether location spoofing is occurring. The decision factors are node credibility and moving track credibility. Fuzzy AHP can reduce the uncertainty of human judgment in detecting LSA and determine the weight of each criterion.

**Fig. 2:**
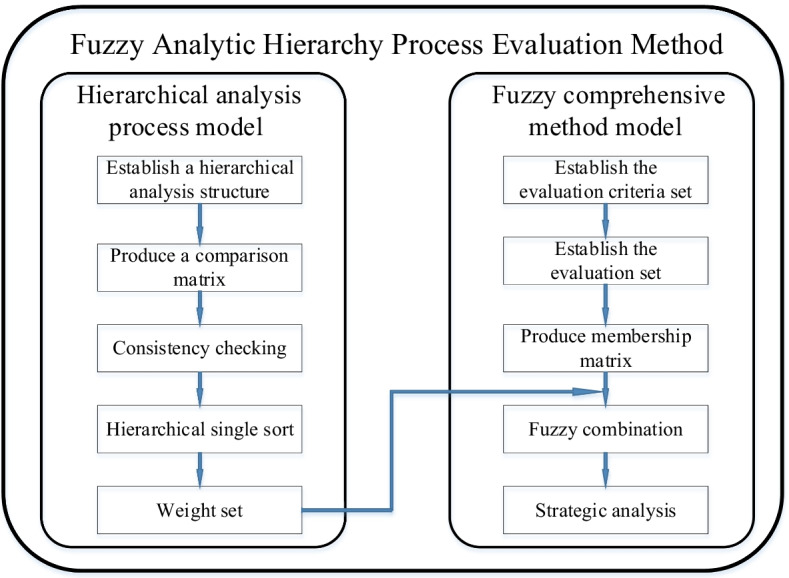
The architecture of FAHP

Based on the architecture of FAHP shown in Fig. 2, the four main steps of the comprehensive evaluation method based on Fuzzy AHP are as follows.

- **Stage 1: Calculate the weight of each criterion based on the AHP**

Priorities are initially derived for the importance of each goal by an expert team, and then adjusted according to the results of experiments. After structuring the comparison matrix, the next step is to calculate the relative weights, and make a consistency check. To calculate the eigenvector using the geometric average, it is necessary to calculate the product of all elements in each row of a pairwise judgment matrix, A, where n is the number of factors at each level.

1 $$M_{i}^{k} = \mathop \prod \limits_{i = 1}^{n} a_{ij}^{k} ,\quad i = 1,2, \ldots ,n$$

2 $$\overline{W}_{i}^{k} = \sqrt[n]{{M_{i}^{k} }}$$

Eigenvectors are calculated and normalized with (3),

3 $$W_{i}^{k} = \overline{W}_{i}^{k} /\mathop \sum \limits_{i = 1}^{n} \overline{W}_{i}^{k}$$

where Vector W is the approximate value of the desired feature vector, which is the weight vector of the relative importance of each factor required.

- **Stage 2: Establish an evaluation set**

In fuzzy evaluation, the evaluation set is composed of a group of fuzzy terms such as ‘high’, ‘medium’, and ‘low’. The ultimate goal of fuzzy AHP evaluation is to produce the best possible evaluation results based on a comprehensive consideration of the various factors involved. In this study, a reasonable evaluation grade is given for the final proof of location (PoL) credibility in the blockchain system by considering the measured data.

- **Stage 3: Calculation of fuzzy comprehensive evaluation matrix**

In fuzzy mathematics, a number between 0 and 1 is used to describe the fuzziness, indicating the degree of correspondence between two concepts. In the process of evaluation of location spoofing, it is necessary to determine the membership degree expression according to the influence of various factors on the evaluation of the credibility of the PoL. Then the membership degree is obtained from the measured data. The membership function is a function in which the membership degree changes with changes in the factors. After the evaluation set is determined, we can select the appropriate membership function, and write the membership degree in a matrix form.

- **Stage 4: Fuzzy evaluation method**

A fuzzy comprehensive evaluation vector can be obtained by multiplying the AHP weight set and the membership degree matrix. Each element in the result represents the membership degree of the evaluation object to the corresponding level in the evaluation set after considering all factors. The final evaluation grade can be obtained by using the weighted average fuzzy comprehensive algorithm to deal with the fuzzy comprehensive evaluation vector.

Due to the complexity of objective things, and the difference in the experiences of experts, a judgment matrix cannot have complete consistency. To investigate whether a judgment matrix can be used, it is necessary to check the consistency of the judgment matrix. In AHP, a consistency index (CI) is defined to measure the inconsistency within the pairwise comparison matrix.

4 $$CI = \frac{{\lambda_{\max } - n}}{n - 1}$$

The consistency ratio (CR) is used to measure the degree of C.I. using the following equation. Where CI is calculated using (4), and the RI value is Random Index that shown in Table 2.

5 $$CR = \frac{CI}{{RI}}$$

**Table 2:** Average random consistency index RI

*n*	1	2	3	4	5	6	7	8	9	10
RI	0	0	0.52	0.89	1.12	1.26	1.36	1.41	1.46	1.49

In general, with a decrease in CR value, the consistency of the judgment matrix will improve. If the CR value is less than 0.1, the inconsistency degree of the comparison matrix A is considered acceptable, and the eigenvector can be used as the weights of the criteria. Otherwise, the comparison matrix needs to be adjusted.

## Evaluation criteria for blockchain proof of location based on fuzzy AHP

Due to the characteristics of the blockchain database, which is comprehensively recorded and cannot be deleted, there is much valuable historical information in the blockchain system. Although the location information is not as accurate as that provided by GPS or other systems, the credibility of user current location information can be proved by witness nodes. Before arriving at a location, every witness node that the user passes will generate location proof information and store it in the blockchain. This historical information can be regarded as the basis of the user's reputation in the blockchain system. Unlike the signal or sensor parameters used by traditional location attestation systems, detection of location spoofing in the blockchain system pays more attention to the historical information of users and witnesses. To sum up, this paper divides the judgment criteria into two aspects: the node and the moving track, and the weight of each criterion is determined by an expert group. The Fuzzy AHP algorithm is used to judge the position generated by the user and a node, and to evaluate its credibility.

**Fig. 3:**
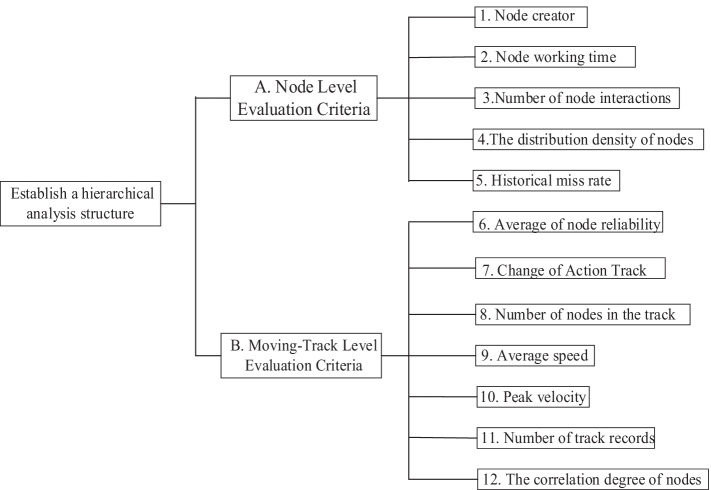
The hierarchical evaluation criteria

The fuzzy analytic hierarchy process (FAHP) has been applied in many fields, because it can rank choices according to the order of their effectiveness at meeting the objectives. This phase includes the establishment of an appropriate AHP model hierarchy for reliable analysis of certain suspicious nodes and users. In general, trust is based on the analysis of multiple messages about the same event, or is accumulated gradually from positive records in the user's moving-track when applying the FAHP technique to construct a more detailed evaluation system based on trust. We assumed that the node-level criteria are as important as track-level criteria in determining whether a user has LSA behavior. We divided the criterion layer into two layers. Since part of the criteria at the moving-track level are based on the evaluation results of the witness nodes included in the moving-track, the formalization of the architecture combining the two types of criteria is an issue. With the Delphi Method, a consensus among experts can be reached, to establish a hierarchical structure for the evaluation of location spoofing. The hierarchy is defined as follows shown in Fig. 3.

### Node level evaluation criteria

#### Node creator

The identity of the creator is the most basic credibility proof of the node. Depending upon the identity of the organization or individual that created the node, the credibility of the node in the blockchain system will change. We expect organizations such as governments and businesses to place as many BLE witness nodes as possible, which may reduce the probability of detecting false or problematic nodes arranged by malicious users. Therefore, we divided the creators into four grades according to the evaluation criteria of the AHP algorithm, with the degree of trust decreasing from government, to organization, to individual, to anonymous.

#### Node working time

A witness node that works normally in the blockchain system for a long time is also considered reliable. Once there is a false BLE node in the area, it will inevitably disappear from the moving track records of many normal users. When these nodes are constantly missing users from their coverage area, or reporting incorrect information, they will not be able to accumulate the qualified working time recorded in the blockchain system. In this evaluation standard, the trust weight of nodes increases with the increase in working time, in a way similar to the logarithmic function. When the working time increases from 0, the weight score increases greatly. When the working time exceeds a set value, further increase has little impact on the node.

#### Number of node interactions

When an interaction between a BLE witness node and a verifier is frequent, the Bluetooth device of the node is assumed to be working normally. This frequency also indicates that the flow of people in the area covered by this node is large. We expect witness nodes to be distributed along roads or in busy areas, so the nodes in these locations are more important in the blockchain system. In the evaluation criteria based on AHP, the impact trend of the number of node interactions on node reputation is similar to that of the node working time.

#### Distribution density of nodes

Since the Bychain system applies the virtual potential field-based excitation assignment algorithm; malicious users usually deploy fake nodes in an uncovered area to cheat the blockchain for rewards. With a higher distribution density of nodes in a region, these nodes are more likely to be considered deployed by trusted users. If a node failure affects the operation, the system can still provide LBS for users passing through this area via other nodes.

#### Historical miss rate

The historical miss detection rate of a node refers to the proportion of the decrease in the number of location proofs provided by the node over time, compared with the average number of adjacent nodes. A BLE witness node may not be guaranteed to interact with every nearby user, but its historical miss rate should be kept low. In the evaluation system, the credibility of nodes with a high historical missed detection rate is reduced.

The above five evaluation criteria at the node level reflect the trustworthiness of a witness node in the blockchain system from different perspectives. BLE nodes that have worked only a limited number of times since deployment, or that make collusion attack with the prover, will be limited in their credibility in the system. It is difficult for malicious users to deploy fake witnesses that meet the above evaluation criteria in reality.

### Moving-track level evaluation criteria

#### Average of node reliability

In the evaluation index system of a location spoofing detection system based on the AHP algorithm, the user moving track information stored in the blockchain is also a major basis for judgment. Among the multiple evaluation criteria at the level of moving track, the most important one is the credibility of all the witness nodes in the track. Through the node level evaluation index system introduced in the previous section, the credibility of a single witness node is obtained based on the AHP algorithm, and the average confidence of the nodes in the moving-track can be calculated. The historical track does not include the node that the user is currently on.

#### Change of action track

The analysis of this indicator is based on the historical track data of users with similar activity routes, as recorded in the blockchain. In a real situation, people usually choose the most convenient route to a target location, taking into account road distribution, urban planning, and the obstructions caused buildings. The most frequent track in the record may be the most reasonable route. However, a false moving track fabricated by malicious users may be quite different, or even impossible, to implement in real life. Once the user's track is different from the track often selected by users at the same start and end positions, the score of the evaluation standard of the track will be reduced.

#### Number of nodes in the track

This evaluation criterion focuses on the length of the user's current moving track to verify users’ location claims. Before reaching the coverage area of the current node, the more interactions between the user and the node, the higher the score in the evaluation system. When the number of nodes in the track is greater than 20, the quantization value is 1.

#### Average speed

The exact time and location of interactions between a verifier and a witness node are recorded in the PoL message. By calculating the distance and elapsed time between two position proofs of users in the blockchain system, the average speed of users can be calculated. Assuming that the distance between two adjacent BLE nodes is close enough, the travel modes used by users can be considered to be unchanged. The common travel modes known include walking, cycling, and by car. The average speed of users should be within the speed range that these three travel modes can achieve.

#### Peak speed

Peak velocity refers to the maximum velocity calculated between adjacent nodes in a moving track. This evaluation criterion is similar to the Average Speed. Once the peak velocity is too large, it will reduce the track's trust score in the evaluation system.

#### Number of track records

Similar to some of the evaluation criteria for nodes, we assume that a user who has been using the blockchain positioning function for a long time is trusted. Compared with newly registered users, long-term users of Bychain are less likely to make LSA. Based on this evaluation logic, the user's score in these standard increases with the historical data recorded on the chain.

#### The correlation degree of nodes

This evaluation criterion is based on the characteristics of possible attacks by malicious users. The blockchain system can obtain information about the identity of the node creator. If more than two-thirds of the nodes in a track belong to the same user, these nodes will be considered to be unreliable nodes. Similarly, those nodes that often have signed on the false PoL message identified by the blockchain detection system will also be identified as unreliable nodes. With an increase in the number of unreliable nodes, the score of the node association degree of the track decreases.

From the above discussion, it can be found that AHP-based trust calculation can be employed to correlate trust values at the track level with the user's proof of location reputation. The mechanism can take into account many kinds of information for the calculation of the trust values. If a malicious user wants to forge a false track record by invading multiple witness nodes in the blockchain system, the evaluation index system established by the above evaluation criteria at the track level will make the LSA difficult to achieve. The cost of spoofing the detection system with fake proof of location would outweigh the possible benefits, forcing malicious users to give up collecting rewards.

## Results and discussion

The above discussion introduced the criteria for evaluation of the credibility of a user's proof of location in the blockchain. After selecting the evaluation criteria according to the needs of the location spoofing detection system, the weight of each sub-criterion on the main criteria is obtained using a judgment matrix provided by experts. Only the correct value of the analysis matrix can reach a better conclusion. The structure of the location spoofing detection model is shown in Fig. 2.

### Weight analysis

According to the hierarchy, we can set up a matrix between the target layer and the criterion layer. Since a matrix can be subdivided into deeper layers, it is necessary to set up the matrix between the main criteria and sub-criteria. Based on the conclusions of different experts, an expert scoring method is used to construct the judgement matrix and calculate the weight of different criteria. Table 3 shows the judgment matrix of the main criterion layer. Tables 4 and 5 show the judgment matrix of each index in the sub-criteria layers.

**Table 3:** Pairwise comparison of main criteria

Criterion	A	B
A	1	2
B	1/2	1

**Table 4:** Pairwise comparison of sub-criteria

Criterion	1	2	3	4	5
1	1	3	4	1/4	6
2	1/3	1	2	1/3	5
3	1/4	1/2	1	1/5	7
4	4	3	5	1	5
5	1/6	1/5	1/7	1/5	1

**Table 5:** Pairwise comparison of criteria

Criterion	6	7	8	9	10	11	12
6	1	1/5	1/3	1/5	1/2	1/8	1/3
7	5	1	5	3	9	3	3
8	3	1/5	1	1/6	3	1/2	2
9	5	1/3	6	1	5	3	7
10	2	1/9	1/3	1/5	1	1/7	1/5
11	8	1/3	2	1/3	7	1	1/3
12	3	1/3	1/2	1/7	5	3	1

After the pairwise comparison matrix is obtained, local priorities are calculated by solving for the eigenvector of the pairwise comparison matrix. The commonly used methods for calculating eigenvectors include the power method, addition method, and square root method. We chose the arithmetic average method to calculate the weight vector. The result is shown as follows:

6 $$\begin{aligned} \omega_{1} & = [\begin{array}{*{20}c} {0.667} & {0.333} \\ \end{array} ], \\ \omega_{2} & = [\begin{array}{*{20}l} {0.254} \hfill & {0.146} \hfill & {0.117} \hfill & {0.442} \hfill & {0.042} \hfill \\ \end{array} ], \\ \omega_{3} & = \left[ {\begin{array}{*{20}c} {0.033} & {0.328} & {0.083} & {0.266} & {0.034} & {0.139} & {0.117} \\ \end{array} } \right], \\ \end{aligned}$$

If, according to consistency checking, the discriminant matrix is consistent, then the result can be acceptable.

### Evaluation set and definition of membership degree

The evaluation set is a qualitative description of the quality of the evaluation object, and covers all levels of indicators. The specific setting can be determined according to the actual situation and the size of the calculation. The evaluation object in this study is the credibility evaluation of the location proof in the blockchain system, so the evaluation set shown in Table 6 is established.

**Table 6:** Evaluation set

Level	1	2	3	4
Credibility evaluation	Low	Medium	High	Very high
Value	0–0.25	0.25–0.5	0.5–0.75	0.75–1

The membership functions of node-level evaluation criteria and moving-track level evaluation criteria in Sect. 5 are established according to the membership definition of the fuzzy comprehensive evaluation method.

#### Node creator

In the blockchain system, the authentication of the identity of the node creator has four levels: government, organization, individual, and anonymous. We use $A$ as the symbol for the reputation level of the creator. When the identity of the creator is the government, $A$ is 4; when the identity authentication is anonymous, $A$ is 1. The membership function of this criterion is defined as:

7 $$X_{1} = \frac{A}{4}$$

#### Node working time

The credibility of the witness node increases with the increase in working time. $B$ is the effective working time of the current BLE node obtained from the historical data recorded on the chain, and ${B}_{0}$ is the average working time of the top 50% of nodes in the blockchain system. The membership function of this criterion is defined as:

8 $$X_{2} = \left\{ {\begin{array}{*{20}l} {\frac{{\log_{10} (B + 1)}}{{\log_{10} \left( {B_{0} + 1} \right)}}} \hfill & {B < B_{0} } \hfill \\ 1 \hfill & {B \ge B_{0} } \hfill \\ \end{array} } \right.$$

#### Number of node interactions

The number of interactions of the current node is $C$. ${c}_{0}$ is the average interaction times of witness nodes in the blockchain system. The membership function of the node interaction times is as follows:

9 $$X_{3} = \tan^{ - 1} \left( {\frac{C}{{c_{0} }}} \right) \times \frac{2}{\pi }$$

#### Distribution density of nodes

Node distribution density refers to the number of nodes in a region. According to the number of adjacent nodes around a witness node, the node distribution density, $D$, can be divided into ten levels, 0–9. The membership function of this criterion is defined as:

10 $$X_{4} = \frac{D + 1}{{10}}$$

#### Historical miss rate

The blockchain system can calculate the historical miss detection rate, $E$, of BLE nodes using the historical data. According to the definition of the historical missing rate criterion, the membership function of this criterion is defined as:

11 $$X_{5} = \frac{1}{{1 + (10E)^{2} }}$$

#### Average of node reliability

The reliability of a single node in the moving track is obtained by the first five evaluation criteria. Therefore, the average value of track reliability $F$ is 0–1, and the specific value is determined by each node in the track.

#### Change of action track

This criterion focuses on the number of different nodes between the current user's moving track and the most common track with the same starting and ending points. $G$ is the proportion of the number of different nodes to the total number in the track. The membership function of this criterion is defined as:

12 $$X_{7} = \frac{1}{{1 + (10G)^{2} }}$$

#### Number of nodes in the track

H is the number of witness nodes in the moving track of the prover. The membership function of this criterion is defined as:

13 $$X_{8} = \tan^{ - 1} \left( \frac{H}{5} \right) \times \frac{2}{\pi }$$

#### Average speed

A moving track is composed of small distances between each node in the blockchain system. The average speed between adjacent nodes is matched with the reasonable speed of different traffic modes, and the credibility weight of each distance is obtained. $I$ is the average of all sub-weights in the track.

#### Peak velocity

$J$ is the maximum velocity between two adjacent nodes in the prover's track the membership function of the criterion is defined as:

#### Number of track records

$K$ is the number of user history tracks recorded in the blockchain. The membership function of this criterion is defined as:

14 $$X_{11} = \tan^{ - 1} (K/10) \times \frac{2}{\pi }$$

#### Correlation degree of nodes

$L$ is the proportion of the number of unreliable nodes in a track to the total number of witness nodes. The membership function of this criterion is defined as:

15 $$X_{12} = \frac{1}{{1 + (10L)^{2} }}$$

${X}_{1}-{X}_{12}$ are all between 0 and 1. This value represents the difference between the malicious user's LSA data and the normal data under this criterion. If one value is larger, the impact of the evaluation criteria on the corresponding indicators of location fraud detection is greater. The membership degree of each criterion is calculated from the measured data.

### Fuzzy evaluation matrix and final result

**Fig. 4:**
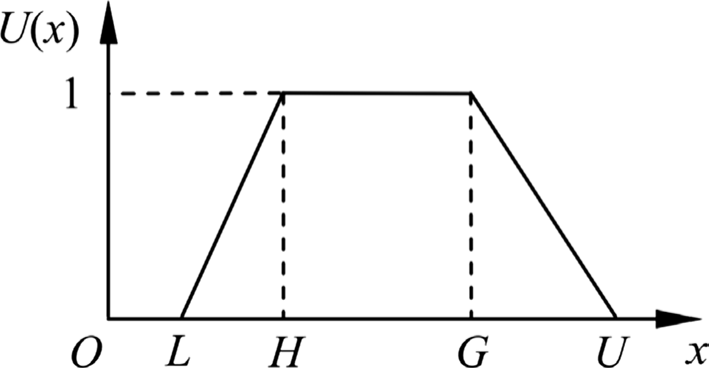
The hierarchical evaluation criteria

Before calculating the fuzzy evaluation matrix, the membership function should be selected according to the actual situation. A fuzzy mapping $f\left({S}_{i}\right): {S}_{i}\to \varphi (W)$ from Membership vector ${S}_{i}$ to evaluation set $W$ is obtained by selecting the appropriate Membership function. The common methods used to determine the Membership function are fuzzy statistics, the three-point method, the fuzzy distribution method, and the expert scoring method. In this study, we used the trapezoidal Membership function in the fuzzy distribution method, to represent subjective pair-wise comparisons of the evaluation process. The parameter $L$, $H$, $G$ and $\mathrm{U}$, respectively, denote the smallest possible value, the average probable values (harmonic mean and geometric mean), and the largest possible value describing a fuzzy event [8]. According to the characteristics of the interval number of the evaluation set, the specific form is as follows

16 $$r_{ij} = \left\{ {\begin{array}{*{20}l} {\frac{{a_{i} }}{{x_{1} }}} \hfill & {0 \le a_{i} \le x_{1} } \hfill \\ 1 \hfill & {x_{1} \le a_{i} \le x_{2} } \hfill \\ {\frac{{1 - a_{i} }}{{1 - x_{2} }}} \hfill & {x_{2} \le a_{i} \le 1} \hfill \\ \end{array} } \right.$$

where ${r}_{ij}$ is the fuzzy matrix element; *a*_*i*_ is a member of the Membership Vector ${S}_{i}$; ${x}_{1}$ and ${x}_{2}$ denotes, respectively, represent the two boundary values of the corresponding interval of the evaluation grade. For example, for the trust level in the evaluation set, the corresponding interval is [0.75 − 1], the value of ${x}_{1}$ is 0.75, and the value of ${x}_{2}$ is 1. From the Membership vector, evaluation set, and Membership function, the sub-criteria evaluation fuzzy consistent judgment matrices are obtained as follows (Fig. 4).

17 $$R_{1} = \left[ {\begin{array}{*{20}c} {0.333} & {0.500} & 1 & 1 \\ {0.520} & {0.74} & 1 & {0.84} \\ {0.253} & {0.380} & {0.760} & 1 \\ {0.187} & {0.280} & {0.560} & 1 \\ 1 & {0.48} & {0.24} & {0.160} \\ \end{array} } \right]$$

18 $$R_{2} = \left[ {\begin{array}{*{20}c} {0.293} & {0.440} & {0.880} & 1 \\ {0.867} & 1 & {0.700} & {0.467} \\ {0.147} & {0.220} & {0.440} & 1 \\ {0.293} & {0.440} & {0.880} & 1 \\ {0.213} & {0.320} & {0.640} & 1 \\ {0.453} & {0.680} & 1 & {0.880} \\ {0.880} & 1 & {0.680} & {0.453} \\ \end{array} } \right]$$

The matrix ${R}_{i}(i=\mathrm{1,2})$ describes the fuzzy evaluation score of the sub-criteria of the main criteria. Based on the comprehensive evaluation model ${B}_{i}={\omega }_{i}\bullet {R}_{i}$, the calculated results are normalized. The secondary evaluation results are as follows.

19 $$\omega_{1} = \left[ {\begin{array}{*{20}c} {0.667} & {0.333} \\ \end{array} } \right],$$

20 $$\omega_{2} = \left[ {\begin{array}{*{20}c} {0.254} & {0.146} & {0.117} & {0.442} & {0.042} \\ \end{array} } \right],$$

21 $$\omega_{3} = \left[ {\begin{array}{*{20}c} {0.033} & {0.328} & {0.083} & {0.266} & {0.034} & {0.139} & {0.117} \\ \end{array} } \right],$$

22 $$B_{1} = \left[ {\begin{array}{*{20}c} {0.315} & {0.423} & {0.747} & {0.942} \\ \end{array} } \right],$$

23 $$B_{2} = \left[ {\begin{array}{*{20}c} {0.557} & {0.700} & {0.770} & {0.744} \\ \end{array} } \right],$$

24 $$B_{0} = \left[ {B_{1} ;B_{2} } \right],$$

25 $$B = \omega_{1} \cdot B_{0} ,$$

26 $$W_{0} = \left[ {\begin{array}{*{20}c} {0.25} & {0.50} & {0.75} & 1 \\ \end{array} } \right].$$

The fuzzy evaluation weight matrix composed of ${B}_{1}$ and ${B}_{2}$ is calculated using formula 17. The main criteria layer evaluation result is calculated using formula 18, and it is normalized to obtain ${B}^{^{\prime}}$= [0.156 0.203 0.297 0.345]. ${W}_{0}$ can be obtained from the grade division and numerical interval of the evaluation set.

27 $$Qi = B^{\prime} \cdot W_{0}$$

In this study, we used the weighted average fuzzy synthesis algorithm. The evaluation result of the POL credibility of the user by the location spoofing detection system was finally calculated using Formula 19. The final score calculated from the sample user data was about 0.71. According to the interval division of the evaluation set, its credibility was high. This result is very similar to that obtained by experts after analyzing the user's data.

To verify the effectiveness of the fuzzy AHP evaluation method, 10,000 randomly selected proof of location sample data were evaluated in the simulation. A neural network method was used to score the samples and compared with FAHP. Calculated values for the data above and the experimental results obtained using the two methods are shown in Table 7. Obviously, the proposed evaluation scheme has a better performance than the neural network [14].

**Table 7:** Evaluation results

Method	FAHP	The neural network
10,000 sample accuracy	9020	8518

## Conclusions

In this work, the fuzzy analytic hierarchy process (FAHP) is used to establish a method for detecting location spoofing in the blockchain based IoT system. By evaluating the credibility of the user's submitted proof of location, this scheme effectively reduces the adverse effects caused by many uncertain factors and human factors. The fuzzy AHP is used to quantify the evaluation results, providing an objective method for detecting fraud perpetrated by malicious users. This approach is conducive to improving the ability of blockchain based IoT systems to defend against LSA. However, due to the complexity and the diversity of the decentralized and permissionless blockchain protocol system Bychain, the establishment of an index system and evaluation method used in this study has some limitations, which need to be further studied.

## Data Availability

Data sharing not applicable to this article as no datasets were generated or analysed during the current study.

## Abbreviations

IoT: Internet of things
AHP: Analytic hierarchy process
SRC: Short-range communication
AI: Artificial intelligence
LBS: Location-based services
SRC: Short-range communication
PoL: Proof of location
LSA: Location spoofing attack
ELSA: Enhanced location spoofing detection algorithm
SLOT: Secure location of things
VANETs: The vehicular ad hoc networks
BLE: Bluetooth low energy
FAHP: The fuzzy analytic hierarchy process
